# Magnetic Resonance Elastography of Rodent Brain

**DOI:** 10.3389/fneur.2018.01010

**Published:** 2018-11-27

**Authors:** Mathilde Bigot, Fabien Chauveau, Olivier Beuf, Simon A. Lambert

**Affiliations:** ^1^Univ. Lyon, INSA-Lyon, Université Claude Bernard Lyon 1, UJM-Saint Etienne, CNRS, Inserm, CREATIS UMR 5220, U1206, Lyon, France; ^2^Univ. Lyon, Lyon Neuroscience Research Center, CNRS UMR 5292, INSERM U1028, Univ. Lyon 1, Lyon, France

**Keywords:** magnetic resonance elastography, brain, neurodegenerative diseases, rodent, MRI

## Abstract

Magnetic resonance elastography (MRE) is a non-invasive imaging technique, using the propagation of mechanical waves as a probe to palpate biological tissues. It consists in three main steps: production of shear waves within the tissue; encoding subsequent tissue displacement in magnetic resonance images; and extraction of mechanical parameters based on dedicated reconstruction methods. These three steps require an acoustic-frequency mechanical actuator, magnetic resonance imaging acquisition, and a post-processing tool for which no turnkey technology is available. The aim of the present review is to outline the state of the art of reported set-ups to investigate rodent brain mechanical properties. The impact of experimental conditions in dimensioning the set-up (wavelength and amplitude of the propagated wave, spatial resolution, and signal-to-noise ratio of the acquisition) on the accuracy and precision of the extracted parameters is discussed, as well as the influence of different imaging sequences, scanners, electromagnetic coils, and reconstruction algorithms. Finally, the performance of MRE in demonstrating viscoelastic differences between structures constituting the physiological rodent brain, and the changes in brain parameters under pathological conditions, are summarized. The recently established link between biomechanical properties of the brain as obtained on MRE and structural factors assessed by histology is also studied. This review intends to give an accessible outline on how to conduct an elastography experiment, and on the potential of the technique in providing valuable information for neuroscientists.

## Introduction

Elastography is an imaging technique which derives mechanical property maps from a visualization of shear waves propagating within soft biological tissues. Originally developed in 2D using ultrasound imaging (1), elastography rapidly came to be associated to magnetic resonance imaging (MRI) methods, which have the advantage of visualizing shear waves in 3D (2). Magnetic Resonance Elastography (MRE) requires several basic experimental steps: (i) a mechanical actuator to generate shear waves within the biological tissue; (ii) encoding of consequent tissue displacements in the phase of MR images, using Motion Encoding Gradients (MEG); and (iii) inversion of the motion equation for each voxel of the images, using a dedicated reconstruction method.

Elastography provides similar information to palpation, and the mechanical properties measured *in vivo* are derived from rheological models. Compared to other MRI methods,

MRE-derived parameters provide the largest “variations […] over 5 orders of magnitude among various physiological states” (3), making MRE sensitive to various kinds of microscopic change (4), and turning MRE into a unique non-invasive tool able to probe tissue at a microscopic scale (5, 6). In the last decade, MRE has shown itself particularly useful for studying the brain, as both palpation, and ultrasound elastography are prevented by the presence of the skull. Several studies in humans reported physiological differences between brain areas, along with alterations in viscoelastic parameters in various diseases (tumor, hydrocephalus and neurodegenerative disease) recently reviewed in (7, 8). These proof-of-concept studies motivated application of MRE in rodents as a crucial step to identify the pathophysiological processes underlying changes in viscoelasticity (9). As of today, this powerful non-invasive technique has provided first *in vivo* mechanical characterizations of healthy and pathological rodent brain even though disparity in results can be pointed out. The goal of this review is to present pros and cons of the diverse actuation, imaging, and inversion methods developed so far as well as some important results drawn out in the field of rodent brain MRE, in order to identify the remaining obstacles to pulling this field out of the hands of expert only.

## *in vivo* rodent brain MRE

### Dimensioning MRE experiments

The accuracy and precision of MRE reconstruction methods inherently depends on the wavelength and amplitude of the propagating wave, the size of the biological structures, and the spatial resolution and signal-to-noise ratio (SNR) of the MRE acquisition. Therefore, as reported in two studies of the quality of MRE-reconstructed data, MRE experimental parameters have to present: a wavelength-to-pixel-size ratio of 15 to 30 (10) or 6 to 10 (11), depending on the reconstruction technique; a minimum phase accrual induced by motion encoding (i.e., wave amplitude) about 10-fold higher than the standard deviation of the phase noise; and, like in morphological MRI, a minimum number of pixels per unit length to image the structure of interest. For rodent MRE, a brain size of about 1 cm implies a working frequency of about 1,000 Hz, i.e., a wavelength of 1 to 2.5 mm. To satisfy the previous wavelength-to-pixel-size ratio criteria, the spatial resolution needs to be in the range of 0.1 × 0.1 to 0.5 × 0.5 mm2. Wave amplitude should range between a few micrometers and 40 μm. Figure S1 illustrates frequency and resolution relationship in human and mice brain.

### MRE transducer

For monochromatic wave propagation in the brain, the rodent head should be kept immobilized, with permanent contact with the actuator. Head locking is usually secured with bite and/or ear bars (9) integrated in the anesthesia mask (12–16). Custom-made head-holders have also been developed: basket-shaped (17) and plaster baskets (18) keeping the animals (young rats or mice) supine to further maximize coupling with the actuator, or 3D-printed plastic neck holders (19, 20).

In the first mouse brain MRE experiments, effective actuator binding was ensured by a nut directly glued to the skull after scalp incision (9). Now, one of the most widespread non-invasive actuation methods avoids the skull (and its associated wave attenuation or reflection) and relies on bite-bars, already used for constraint (13, 14, 21). However, this can provoke bulk motion, diminishing transmission efficiency, and impairing wave quality. It was therefore replaced in certain cases by a simple piston positioned on the top of the head of animals lying supine (17, 18). 3D-printing techniques facilitated the development of new designs. A nose-cone held in place by an elastic band can both deliver gaseous anesthesia and transmit mechanical vibration (19, 20).

Waves can be produced in continuous mode using electromechanical or acoustic transducers (13, 17, 18, 20, 22, 23) in order to establish the mechanical steady-state required to satisfy the motion equation used for reconstruction (14). The magnetic components of such transducers compel their positioning outside the MRI bore. This relatively long distance (50–100 cm, depending on the fringe field) requires high transducer force and minimal friction in order to achieve efficient transmission (20, 22). Some piezoelectric drivers are compatible with MRI and can be placed in close proximity to the animal (5–15 cm) to preserve wave amplitude (14, 21, 24–27).

Finally, electromagnetic transducers have been built based on a shielded solenoid coil positioned with its axis perpendicular to the static magnetic field (9, 16, 28–33). Both piezoelectric and electromagnetic transducers require a wave generator combined with an amplifier.

Actuators are able to generate waves from 100 (13) to 2,500 Hz (34). To increase amplitude, frequencies corresponding to the resonance of the loaded system can be used, determined using accelerometers (13) or vibrometers (25, 26).

**Figure 1:**
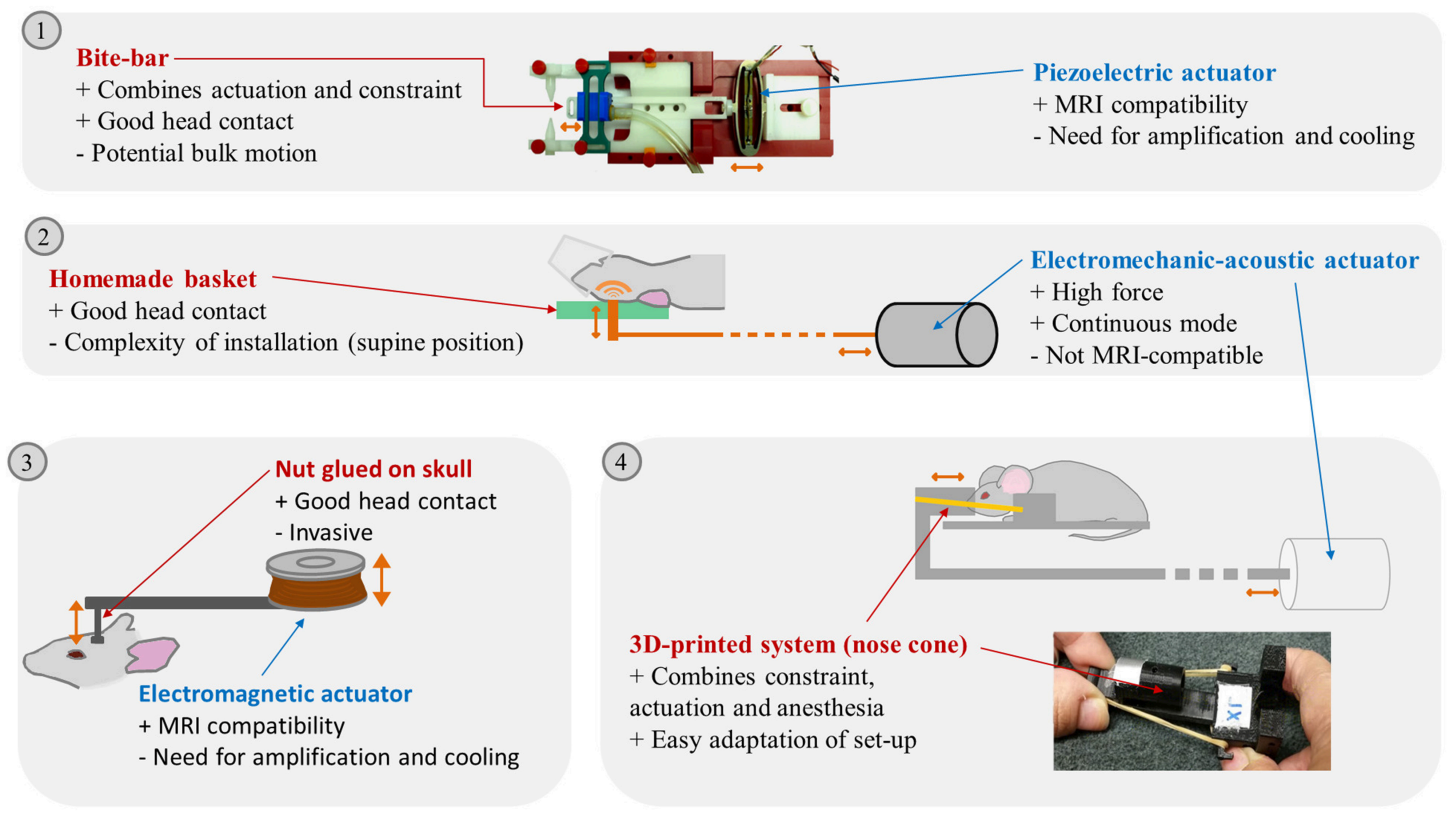
Illustration of different brain MRE set-ups reported in the litterature. (1) Picture of a piezoelectric actuator linked to a bite-bar by a plastic rod, Clayton 2011. (2) schema of a modal actuator driving a vertical piston hitting the back of the rodent head, Chatelin 2016. (3) Electromagnetic actuator driving a vertical piston glued on the mouse skull, Atay 2008. (4) Schema of the external transducer transmitting excitation to a nose cone maintained on the mouse head by rubber bands and picture of the nose cone and plots maintaining the mouse head, Patz 2017. Advantages (+) and drawbacks (–) of the **actuation transmitters** and **actuators** are listed. Actuation movements are symbolized by orange arrows. Pictures extracted from (14) © Institute of Physics and Engineering in Medicine. Reproduced by permission of IOP Publishing. All rights reserved; and (20).

Overall, a large number of actuation devices for MRE have been reported, combined with various constraint and coupling devices. This technological diversity is bound to produce waves with differences in amplitude and relative proportions of shear and compression waves, and artifacts (e.g., wave wrapping at different parts of the brain). For instance, the use of a piezoelectric actuator coupled with an incisor bar was able to generate waves with amplitudes of 1 to 2.5 μm for a frequency range of 600–1,800 Hz for a specific set-up (14), whereas it reached 40 μm at 877 Hz in another set-up (26). A summary of the advantages and drawbacks of the various experimental set-up elements can be found in Figure 1.

In conclusion, a large span of set-ups is reported in the literature, an evolution toward an easy to handle standardized solution could facilitate direct comparison between the results from different teams.

### MRE pulse sequences

MRE sequences are generally based on conventional MRI sequences, but MEGs are added to sensitize movement in the phase image. These MEGs are synchronized at different wave propagation time-points in order to obtain an image at different steps over this propagation period. To avoid other phase encoding (for example, due to static magnetic field) being superimposed on shear wave motion, a straightforward solution is to acquire MRE images without inducing vibration, to be subtracted from the image with vibration (9, 21). Another solution to increase phase-to-noise ratio is to invert the MEG polarity (14, 16, 27–30, 35) of the image to be subtracted. Most of the sequences are spin-echo-based, to avoid signal loss due to magnetic field inhomogeneity (25), but a gradient-echo FLASH sequence has also been used to shorten acquisition time and reduce echo time, especially at low frequency (36). Encoding motion with 2D sequences in the three directions for 8 time-steps takes approximately 1 h, with pixels from 0.2 × 0.2 to 0.55 × 0.55 mm2 and slice thickness of 0.2 to 2 mm. Decreasing acquisition time requires single-slice acquisition, motion encoding in a single direction and a smaller number of time-steps, but impairs reconstruction quality and may limit the reconstruction methods able to be used. A Sample Interval Modulation (SLIM) sequence, simultaneously encoding the three directions of motion with MEGs of different frequencies, has been newly developed, shortening total acquisition time from 51 to 17 minutes, without noticeable change in SNR (21).

In conclusion, reducing acquisition time while preserving SNR is still a challenge in MRE, but new encoding strategies could contribute to a more efficient MRE imaging technique.

### Static magnetic field, gradients, and radio frequency coils

High-field MR scanners offer the possibility of greater spatial resolution and SNR, but at the cost of greater absolute magnetic field inhomogeneity and artifacts. For small-animal imaging, 0.1 (13) to 11.7 T (9) fields have been used. Recently, a 0.5 T benchtop scanner to study *ex vivo* tissue in tube was described, coupled with a preamplifier and amplifier, raising MEG strength to 1.2 T.m^−1^ and motion sensitivity to that of high-field micro-MRE (37). Using low-field MRI scanners does not appear to be a major drawback for MRE imaging, especially when filling factors are maximized. With this in mind, surface coils (19, 20), custom-built birdcages (38), and small RF coils (13) have been used; although no comparisons of SNR have been made between scanners and coils, reconstruction of viscoelastic parameters appeared possible with similar spatial resolution (voxel volume < 0.03 mm^3^) for magnetic fields ≥ 4.7 T. Another obstacle to high frequency MRE can be gradient rise-time. Yasar et al. reported difficulty in working beyond 3.8 kHz as gradients could not reach its maximum strength sufficiently fast and without image artifacts (39).

### Reconstruction

The objective of this section is to give a quick overview of how wave (speed and attenuation) and mechanical parameters (complex shear modulus) can be derived from MRE phase images. Different methods have been developed: wavelength estimation method (40, 41), nonlinear inversion finite-element method (42), or direct inversion (43, 44). The first does not accurately detect interfaces and is not suitable for detection of viscoelastic changes in small areas within the brain. Finite-element-based methods are recent, accurate techniques, but are not widespread as they require large calculation capacity and expertise in numerical computation. Finally, direct inversion is the most widely used method in small-animal studies. A thorough explanation of reconstruction methods can be found in (45, 46). For the sake of simplicity, only the direct inversion method is briefly described below. It consists in two main steps.

#### Extraction of shear wave field from measured phase images

This step consists first in getting rid of phase shifts >2π. This can be done using commercial solutions (9) or unwrapping algorithms previously developed in other contexts (47–49). Next, the first harmonic component of motion u, also called the displacement field, is isolated in the frequency domain with a temporal Fourier transform in order to get rid of the static offset caused by susceptibility and B_0_ inhomogeneity (45). Then, shear waves are extracted from u by calculating the curl operator of the measured displacement field or by applying a high-pass filter to remove low-frequency bulk-waves. The curl-based technique has the disadvantage of being sensitive to noise, and hence greatly depends on SNR and filtering methods.

#### Quantification of shear mechanical properties by solving an inverse problem

The displacement field is linked to the shear mechanical properties of tissues by complex motion equations. A locally homogenous, linear isotropic viscoelastic medium is often assumed. Inversion methods taking account of anisotropy have also been proposed to eliminate possible errors caused by myelin fibers that could propagate waves in a particular direction and thus skew the estimate of mechanical properties (50). To our knowledge, no studies confirmed an effect of these fibers on propagation in mice; however, studies on human brain are beginning to show the interest of such a method, as indicated by Murphy et al. in their review (8). To perform reconstruction, displacement field matrices can be inversed directly, but fit algorithms help to reduce discrepancies in the field.

Various algorithms have been developed to post-process MRE data. However, at different steps of the preprocessing and inversion algorithm, a wide variety of filters (high-pass, bandpass, Gaussian, median, directional) have been used for different purposes (compressional waves suppression, noise reduction, data smoothing and direction weight assessment, respectively). Attributing cut-off frequencies and determining the size of the filter is delicate, and often empirically determined from simulations or phantom experiments, and so may not fit complex living tissues.

Despite this apparent diversity of reconstruction methods (51), standardized solutions such as the Elastography Software Pipeline (52) recently appeared. Some are available online: the multi-frequency wave number recovery (53) and the multi-frequency direct inversion (54) and wavelength estimation and direct inversion methods (55, 56).

Finally, depending on the rheological model used for the reconstruction method, different parameters can be used to characterize the mechanical properties of the tissue. One major consequence is the difficulty of comparing different MRE studies. Most recent studies used the curl-based method, allowing extraction of the complex shear modulus G^*^ (kPa), the real and imaginary parts of which comprise the storage modulus G', representing elasticity, and the loss modulus G”, representing viscosity. Fewer studies have reported wave velocity and attenuation, as these parameters are completely entangled compared to G' and G”, although these are well-known in the ultrasound community, and have direct and intuitive interpretation. Finally the parameters of the spring pot model, μ (kPa) and α (dimensionless), representing frequency-independent elasticity and structure, respectively, can help in inferring structural information from tissues at a microscopic scale (57).

## MRE-derived brain parameters in rodents

### Healthy animals

**Figure 2:**
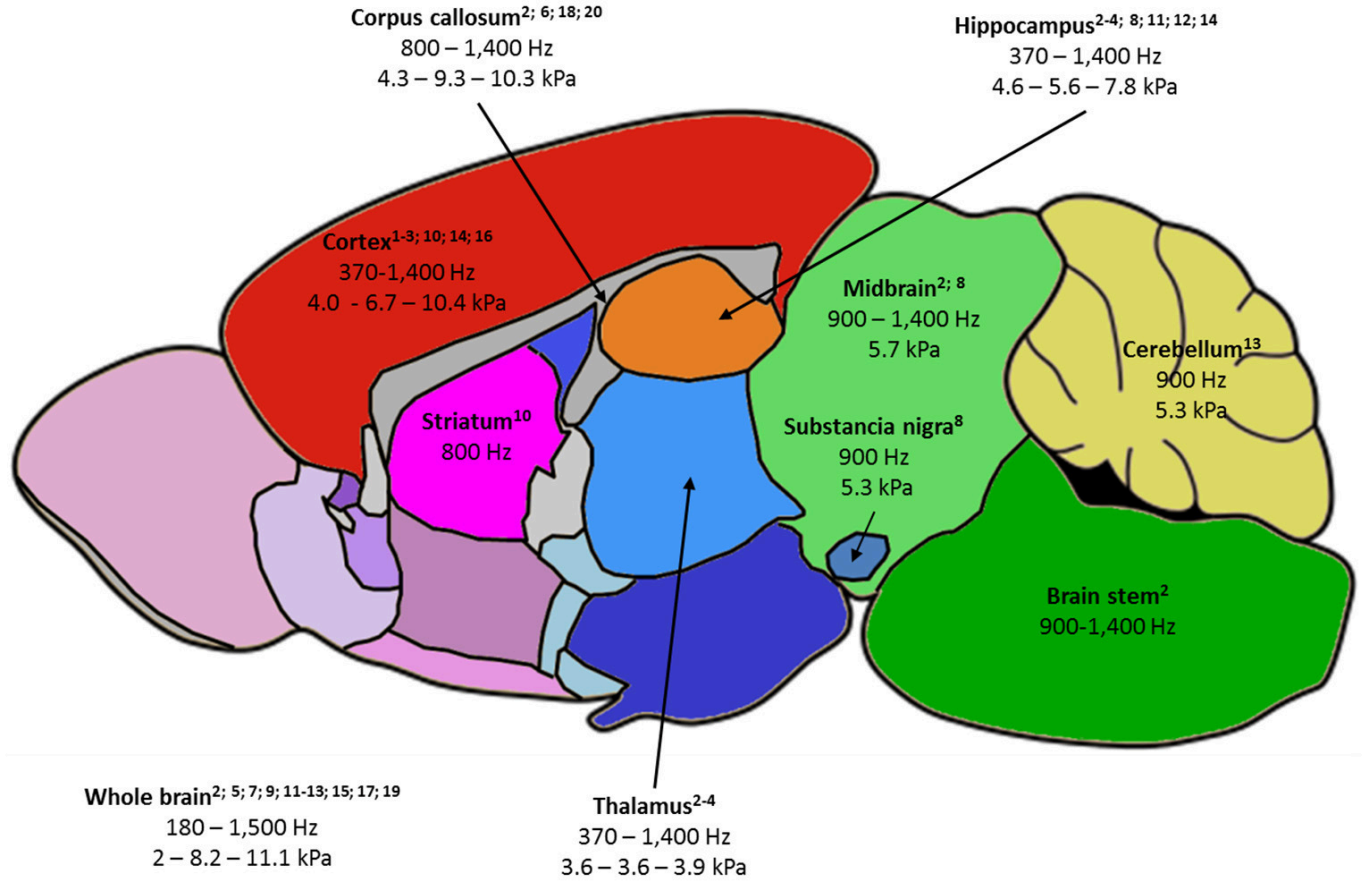
Summary of the rodent brain areas studied by MRE, labeled as follows: -(first line) name of brain area, with relevant publications listed below; -(second line) frequency ranges used to probe the area; -(third line) when available, the minimum value of |G^*^| - the average of |G^*^| values reported at 900 and 1,000 Hz – maximum reported |G^*^| value. |G^*^| was chosen as it is the most fequently reported parameter and 900-1,000 Hz studies the most frequent in the literature. 1—Atay 2008; 2—Bertalan 2017; 3—Boulet 2011; 4—Châtelin 2016; 5—Clayton 2012; 6—Diguet 2009; 7—Freimann 2013; 8—Hain 2016; 9—Jamin 2015; 10—Jugé 2016; 11—Klein 2014; 12—Majumdar 2017; 13—Millward 2015; 14—Munder 2017; 15—Murphy 2012; 16—Patz 2017; 17 – Salameh 2011; 18—Schregel 2012; 19—Vappou 2008; 20—Yin 2017.

Reported values of viscoelastic parameters for healthy animals vary widely. For example, whole-brain magnitude of the complex shear modulus |G^*^| ranges from 2 to 11 kPa (13, 14) and cortical μ, computed for the sake of comparison as indicated by Hiscox et al. (7), from 3 to 13 kPa (13, 30). A first explanation is the large frequency range used (180–1,800 Hz): according to the power-law model (14), loss and conservation moduli are expected to increase with frequency. Other methodological differences in the MRE imaging set-up, reconstruction method, or filters may contribute to these discrepancies. Even though absolute quantitative MRE has not yet been achieved, several reports comparing brain structures showed agreement between animal and human studies: hippocampus was found to be stiffer than thalamus and cortex by Boulet et al. (25), which was confirmed in subsequent studies in rodents (18, 30) and corresponds to human findings (7). Cerebellum was found to be softer than cerebrum in mice (32) and in several human studies (7). Except for one recent study (58), corpus callosum was generally identified as the stiffest area in the brain (17), corroborating findings in humans (59). Generally, structures with higher fiber density appear stiffer (33). Figure 2 summarizes the frequency and |G^*^| ranges reported in healthy rodents. Moreover, MRE demonstrated sensitivity to structural changes occurring with age in maturing rodents (17, 30) and older humans (59).

Finally, agreement with a power-law model has been established for small animals (14), larger animal and humans (60), allowing multi-frequency studies to explore the brain's dispersive properties. These studies could lead to a precise estimate of mechanical parameters independent of frequency [e.g., μ, α and Φ = arctan(G”/G')] and to more accurate elastograms.

### Pathological conditions

Several pathologies affect the biomechanical properties of the brain. In models of traumatic brain injury, both G' and G” were reduced (25, 26). Brain tumors were softer (i.e., less elastic and viscous) than the surrounding parenchyma by a factor 0.6 to 0.8, and biomechanical parameters might help in grading brain malignancies (19, 24, 61, 62). This decrease in both G' and G” could be associated with cell death and decreased microvessel density (62). |G^*^| decrease was of the same order of magnitude (< 40%) in hydrocephalus (22), stroke (28) and multiple sclerosis models (31, 32), while φ remained constant over disease course. This loss was correlated with decreased mean diffusivity on Diffusion Tensor Imaging (22) and with neuron loss on histopathology (28). Reduction in phase angle was associated with demyelination and diminution of |G^*^| with the destruction of the extracellular matrix (17). In mouse models of Alzheimer's and Parkinson's disease, |G^*^| also appeared to drop with the number of neurons (15, 16, 29, 30, 35).

To summarize, MRE appears to be a promising non-invasive tool to diagnose and stage various brain diseases, by probing changes in viscoelasticity. However, multiple pathophysiological processes, such as inflammation, neuron and glial cell density and organization, and vascularization, can potentially affect biomechanical parameters. The link between microstructure revealed by optical microscopy and its impact on the macroscopic mechanical properties of the tissue is delicate to establish as (i) brain microstructure in healthy and pathological states is still not well established (63, 64); the development of quantitative and 3D histological methods and registration pipeline might help furthering recent works aiming to establish brain atlases (65); (ii) Question addressed by Verdier et al. subsists: “Are tissues just a macroscopic generalization of the cell properties?” (4). Comparison between elastograms and other *in vivo* imaging techniques such as DTI or PET are also used to decipher the links between the various parameters evaluated by MRE and the underlying microscopic structure and organization of the brain. Interestingly, vasodilatation caused by activation of cannabinoid receptors showed that blood-flow-dependent tissue softening should not be overlooked when interpreting brain viscoelasticity changes (18). On the other hand, such findings suggest that MRE may also be used for functional brain imaging under a stimulation paradigm (20). Although MRE offers a wide range of potential applications, most exploratory studies published so far have not demonstrated increased sensitivity to specific changes of the tissue microstructure in comparison to other methods. This is a necessary prior step for MRE to be developed as a powerful diagnostic and brain research tool.

## Conclusion

MRE is still a recent imaging technique and lacks standardization at small-animal scale, making reliable quantitative data difficult to obtain. It could benefit from concertation of different MRE experts and, as for human MRE, the development of a commercial solution could help to democratize rodent MRE and ascertain preliminary results. The gold standard for measuring elasticity and viscosity is currently rheometry, which operates at frequencies far lower than those used in small-animal MRE (50–200 Hz vs. 200–1,800 Hz). Phantom and *ex vivo* comparisons with rheometry and simulation are currently the best leads toward establishing a gold standard in MRE, but the use of various homemade elements in the imaging and reconstruction chains, along with an empirical approach to filter setting currently prevent standardization in *in vivo* systems.

Nevertheless, MRE already proved effective in comparing brain structures and physiological and pathological states. Recent studies have begun to unveil the link between brain structure and the changes in various parameters. For example, some MRE studies claim that neuron density is the most important element affecting loss and conservation moduli; this was shown in neurodegenerative disease models, but also in healthy rodents, where the neuron-rich hippocampus is stiffer than the cortex or thalamus. The composition of the extracellular matrix and its disorganization by inflammation or edema can also impact viscoelastic parameters. More detailed comparisons between MRE, histology, microscopy and other imaging techniques are mandatory to disentangle the relationship between mechanical parameters in the brain and biological processes.

## Author contributions

MB, FC, OB, and SL equally contributed to the drafting, analyses, and final version of the whole manuscript. All authors read and approved the final manuscript.

### Conflict of interest statement

The authors declare that the research was conducted in the absence of any commercial or financial relationships that could be construed as a potential conflict of interest.
