# Supplementary material for: Magnetic Resonance Elastography of Rodent Brain
Source: Front Neurol. 2018 Nov 27;9:1010. doi: 10.3389/fneur.2018.01010 (PMC6277573; doi:10.3389/fneur.2018.01010)
Supplement: Supplementary file 1 [file Table_1.docx]

Supplementary Material

Magnetic Resonance Elastography of Rodent Brain

Mathilde Bigot^1^, Fabien Chauveau², Olivier Beuf^1^, Simon A. Lambert^1^*

*** Correspondence:** Simon A. Lambert: Simon.Lambert@creatis.insa-lyon.fr

# Supplementary Figure


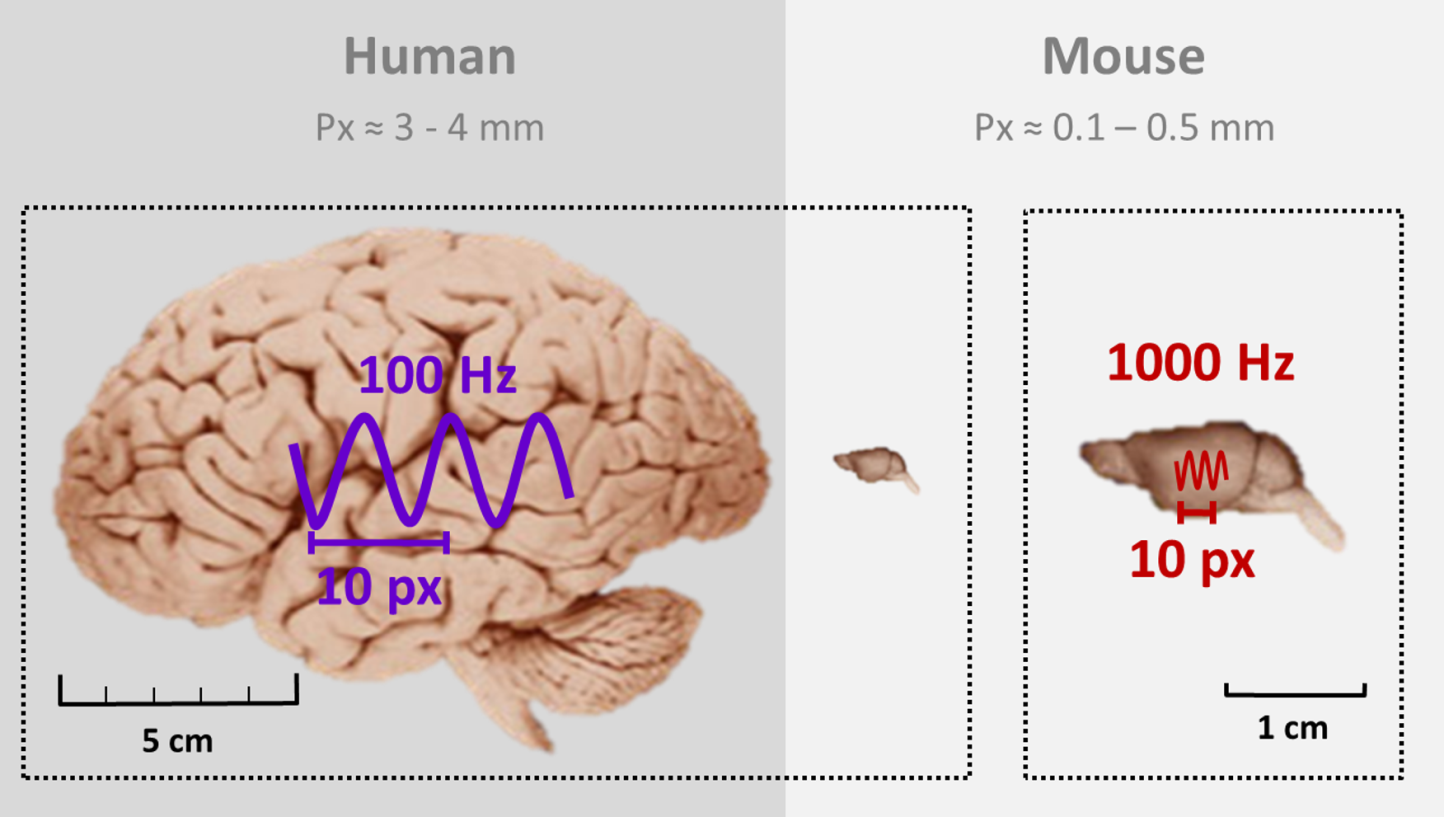


**Supplementary Figure 1.** Images of human (on the right) and murine (on the left) brains. On the human brain and on the zoomed murine brain are represented waves of 100 Hz and 1000 Hz respectively, as well as the average width of 10 pixels. Since the human brain is 10 times larger than the murine brain, the imaging frequency is multiplied by 10 in order to probe the same structures in both species.*[Images from the University and Michigan State Comparative Mammalian Brain Collections and from the National Museum of Health and Medicine. Available at:* [*http://neurosciencelibrary.org/index.html*](http://neurosciencelibrary.org/index.html)*. The preparation of all these images and specimens have been funded by the National Science Foundation and by the National Institutes of Health].*
